# Value of non-coding RNAs to assess lymph node status in cervical cancer

**DOI:** 10.3389/fonc.2023.1144672

**Published:** 2023-05-10

**Authors:** Yohann Dabi, Amelia Favier, Léo Razakamanantsoa, Stéphane Suisse, Yannick Marie, Cyril Touboul, Clément Ferrier, Sofiane Bendifallah, Emile Daraï

**Affiliations:** ^1^ Sorbonne University, Department of Obstetrics and Reproductive Medicine, Hôpital Tenon, Paris, France; ^2^ Clinical Research Group (GRC) Paris 6: Centre Expert Endométriose (C3E), Sorbonne University (GRC6 C3E SU), Paris, France; ^3^ Sorbonne University, Inserm UMR S 938, Centre de recherche de saint Antoine (CRSA), Hôpital Saint Antoine, Paris, France; ^4^ Department of Radiology imaging and Interventional speciality imaging, Tenon Hospital, Paris, France; ^5^ Ziwig, Lyon, France; ^6^ Gentoyping and Sequencing core facility, iGenSeq, Institut du Cerveau et de la Moelle épinière, Institut du Cerveau et de la Moelle (ICM), Hôpital Pitié-Salpêtrière, Paris, France

**Keywords:** cervical cancer, lymph node metastasis, non-coding RNA, microRNA, long non coding RNA, biomarker

## Abstract

Cervical cancer (CC) is the fourth cancer in women and is the leading cause of cancer death in 42 countries. Lymph node metastasis is a determinant prognostic factor, as underlined in the latest FIGO classification. However, assessment of lymph node status remains difficult, despite the progress of imaging such as PET-CT and MRI. In the specific setting of CC, all data underlined the need for new biomarkers easily available to assess lymph node status. Previous studies have underlined the potential value of ncRNA expression in gynecological cancers. In this review, we aimed to evaluate the contribution of ncRNAs in tissue and biofluid samples to determine lymph node status in CC with potential impact on both surgical and adjuvant therapies. In tissue samples, our analysis found that there are arguments to support the role of ncRNAs in physiopathology, differential diagnosis from normal tissue, preinvasive and invasive tumors. In biofluids, despite small studies especially concerning miRNAs expression, promising data opens up new avenue to establish a non-invasive signature for lymph node status as well as a tool to predict response to neo- and adjuvant therapies, thus improving management algorithm of patients with CC.

## Introduction

Cervical cancer (CC) is the fourth cancer in women and is the leading cause of cancer death in 42 countries (1). World Health Organization (WHO) estimated that 570 000 new cases are diagnosed per year causing 311 000 deaths in 2018, with 90% of them observed in low-income and middle-income countries (1, 2). CC is linked to infection by high-risk human papillomavirus (HPV) genotypes but almost 90% of incident HPV infections are cleared within the two years after initial infection and persist only in 10% of women (3–5). However, a debate persists on whether the virus is completely cleared from basal cells or remains latent with potential reactivation. Moreover, it is important to note that the development of cancer takes 10 to 30 years from preinvasive to invasive tumor underlining the crucial role of prevention policies (6). Primary policy is based on HPV vaccination launched in 2006 with three HPV vaccines currently available (bi, quadri, and nonavalent) highly efficient against high-risk HPV 16/18 and 31/33/45/52/58 genotypes causing 70% and 18.5% of all CC, respectively (5). Secondary prevention consists in the detection and treatment of cervical intra-epithelial neoplasia (CIN). This screening is based on Pap smears at regular intervals imposing substantial medical resources while exhibiting a poor sensitivity, and lack in quality assurance results (7). In contrast HPV-based screening has high sensitivity and accuracy and better reproducibility explaining that most of national and international guidelines recommend primary HPV-based screening over conventional Pap smears (8).

Since the 2018 revised classification of the International Federation of Gynecology and Obstetrics (FIGO), imaging and pathological findings have been incorporated in CC staging (**Table 1**) (4). The FIGO stage is directly corelated to prognosis ranging from almost 100% 5-year disease-free survival rates for stage IA to 5–15% for stage IV. Moreover, FIGO stage allows determining patients’ allocation in different treatment regimens (26). In addition to classic CC prognostic factors including lymphovascular space invasion (LVSI), age, comorbidities (e.g., anemia, HIV infection), high-risk histological subtypes such as adenosquamous and neuroendocrine carcinomas that are easily available on biopsy and conventional imaging, lymph node status remains relatively difficult to evaluate (27–29). This issue is crucial as for early stages the risk of lymph node metastasis (LNM) is approximately 3.7 to 21.7%, and the 5-year overall survival (OS) decreases from 80% to 53% in case of LNM+ (30–33). Therefore, there is a need of adequate tools to assess pelvic and paraaortic LNM not only for early but also for locally advanced CC (IB3-IV stages). Conventional MRI and CT are widely recommended although both exhibited low sensitivity and specificity; 37–71 and 83–93, and 31–58 and 92–97, respectively (34, 35). Despite the use of specific MRI sequences, its low accuracy could be related to size and morphologic criteria used to diagnose LNM. In this specific context, previous studies demonstrated higher relevance of FDG PET-CT and FDG PET-MRI with respective sensitivity and specificity of 34–82 and 93–100, and 83–91 and 90–94 (36). However, PET-CT has also some limits as its ability to detect metastatic lymph nodes is depending on lymph node size with respective sensitivity of 100%, 67%, and 13% for metastatic nodes ≥ 10 mm, 5–9 mm, and ≤ 4 mm (37). Although the rate of lymph node involvement is about 20% even for locally advanced CC, these limits explain why recent ESGO-ESTRO-ESP guidelines recommend paraaortic lymph node dissection in locally advanced CC in patients with negative paraaortic lymph nodes on imaging due to its major impact on therapies (38).

**Table 1:** Series evaluating the correlation between miRNAs and lymph nodes invasion in patients with cervical cancer.

Author	microRNA	Down/Up regulated	Number of CC	LNM -	LNM +	p-value
Chen (9)	Mir-508; miR-509-2; miR-526b	Down	145	113	32	<0.05
Barquet Munoz (10)	miR-487b; miR-29b-2-5p; miR-195	Down	25	13	12	NA
	miR-92b-5p; miR-483-5p; miR-4534; miR-548ac	Up				
Wang (11)	miR-186-5p	Down	72	NA	NA	NA
Liu (12)	miR-543	Down	69	38	31	< 0.03
Wei Dong (13)	miR-337	Down	49	20	21	0.013
Chei Fei Zhou (14)	miR-221-3p	Up	107	64	43	< 0.001
Jianbing Liu (15)	miR-205	Up	48	23	25	0.008
Min Luo (16)	miR-26b	Down	88	22	34	0.036
Ying Han (17)	miR-21-3p; miR-21-5p	Up	30	25	5	0.015
Ryunosuk Kogo (18)	miR-218	Down	79	40	39	0.053
Changyan Liang (19)	miR-433	Down	65	35	30	0.001
Long Huang (20)	Let-7c; miR-100; miR-125b; miR-143; miR-145; miR-199a-5p	Down	44	30	14	NS
Li Wang (21)	miR-99a; miR-99b	Down	20	12	8	< 0.05
M Li (22)	miR-142-3p	Down	173	121	52	0.005
J Zhou (23)	miR-1254	Down	181	114	67	0.004
Yanxia Chen (24)	miR-143	Down	77	43	16	0.022
Y Q Wei (25)	miR-9-5p	Up	44	22	22	< 0.001

CC, Cervical cancer; LNM, Lymph node metastasis; NA, Not Available; NS, Not significant.

In the specific setting of CC, all data underlined the need for new biomarkers easily available to assess lymph node status avoiding surgical risks even if para-aortic lymphadenectomy is feasible by mini-invasive surgery.

Numerous studies support that non-coding RNAs (ncRNA) are essential for tumorigenesis by regulating the expression of tumour-related genes. ncRNAs can regulate gene expression primarily by acting as transcription factors, regulating chromatin remodelling or participating in post-transcriptional regulation (39). Moreover, ncRNAs act by guiding DNA synthesis or gene rearrangement, and protecting the genome from foreign nucleic acids (40). In gynaecological cancers, previous studies have underlined the potential value of ncRNA expression in tissue samples to assess prognosis and especially lymph node status in endometrial carcinoma (41). Among ncRNA, miRNAs have been extensively studied showing that they can function as oncogenes and/or tumor suppressor genes depending on the function of their target genes. For CC, although abnormal expression of miRNAs has been demonstrated to be linked to cancer biology, including proliferation, differentiation, apoptosis, migration, invasion, and metastatic angiogenesis (42–49), few reports focused on their relevance to determine lymph node status. Moreover, some studies support that miRNAs emerge as potential tools to differentiate benign from preinvasive and invasive tumours and to determine prognosis (50, 51).

Therefore, the aim of the present review was to evaluate the contribution of ncRNAs in tissue and biofluid samples to determine lymph node status in CC with potential impact on both surgical and medical therapies such as brachy and chemotherapy (52).

## ncRNAs

ncRNAs represent about 98% of the transcriptome. Among ncRNAs, arbitrarily those with less than 50 nucleotides are defined as small RNAs (sncRNAs) including microRNAs (miRNAs), Piwi interacting RNAs (piRNAs), transfer RNAs (tRNAs), small nuclear RNAs (snRNAs), and small interfering RNAs (siRNAs) (53, 54). ncRNAs with more than 200 nucleotides are defined as long RNAs (lncRNAs), although an overlapping in nucleotide length exists between snc- and lncRNAs, including intergenic ncRNAs (lincRNAs), some circular RNAs (circRNAs), and ribosomal RNAs (rRNAs) (55).

## miRNA

miRNAs are small intracellular RNAs, 18-25 nucleotides long, capable of inducing the extinction (silencing) of gene expression by post-transcriptional regulatory mechanisms, by binding in a targeted manner to the 3’ untranslated parts (3’UTR) mRNAs thus causing translational blockage or degradation (56). However, another mechanism is binding miRNAs to the 5’UTR regions inducing either activation or repression of translation.

### miRNA expression in CC tissue samples

In contrast to an abundant literature on miRNAs implicated in the pathogenesis and signaling pathways involved in CC, less data is available allowing correlating their expression with lymph node status (57–60). Moreover, most series focused on one or a limited panel of miRNA mainly using paired tissue samples comparing the expression between CC tissue and adjacent normal appearance tissue (**Table 1**).

Using The Cancer Genome Atlas (TCGA) database, Chen Q et al, analyzed the expression of 422 miRNAs in 145 patients with early-stage CC (32 LNM+ and 113 LNM−) showing that 75 miRNAs were differentially expressed between the groups (9). After multivariate analysis, miRNA-508, miRNA-509-2, and miRNA-526b were associated with LNM+ status with target genes implicated in the MAPK, cAMP, PI3K/Akt, mTOR, and estrogen cancer signaling pathways. In the same way, using TCGA database on a small series, Y.Q. Wei et al. among 14 miRNAs differentially expressed in CC with LNM+ (miRNA-9-3p, miRNA-191-5p, miRNA-9-5p, miRNA-873-5p, miRNA-378a-3p, miRNA-624-5p, miRNA-149-5p, miRNA-425-5p, miRNA-519a-5p, miRNA-375, miRNA-151b, miRNA-874-3p, miRNA-92b-5p, miRNA-3605-3p) evaluated the relation between miRNA-9-5p and found a significant overexpression (p=0.0002) in patients with LNM+ (25).

Recently, in a small study using microarray, Barquet-Munãoz et al. reported 36 miRNAs differentially expressed between patients with and without LNM including, 17 over-, and 19 underexpressed miRNAs (10). Among them, 10 exhibited high fold change (FC); miRNAs, miR-487b (FC = −3.2, *p* = 0.0003), miR-194 (FC = −2.8, *p* = 0.006), miR-34c-5p (FC = −2.46, *p* = 0.007), miR-29b-2-5p (FC = −2.3, *p* = 0.007), and miR-195 (FC = −2.07, *p* = 0.001), miR-548ac (FC = 2.74, *p* = 0.0003), miR-4534 (FC = 2.47, *p* = 0.001), miR-483-5p (FC = 2.21, *p* = 0.002), miR-564 (FC = 2.01, *p* = 0.006), and miR-92b-5p (FC = 1.82, *p* = 0.005). This biological miRNA signature allowed to correctly classify 91.6% patients with LNM (11/12) and 92.3% patients without LNM (12/13). However, after qRT-PCR, only seven miRNAs were validated. In a series of 44 CC, Long Huang et al, reported a decrease of a panel of six miRNAs (let-7c, miRNA-100, miRNA-125b, miRNA-143, miRNA-145 and miRNA-199a-5p) correlated with FIGO stage but not with LNM. In a series of 79 CC, R. Kogo et al. reported only a trend for a lower miRNA-218 expression in patients with lymph node metastasis (p=0.053). However, the authors reported a relation between a decrease in miRNA-218 and the occurrence of lymph node recurrence both in pelvic (p=0.032) and para-aortic areas (0.013) (18). In a series of 72 CC vs adjacent normal tissue, A. Wang observed that miR-186-5p was down regulated in patients with LNM+ but with missing data on CC characteristics (11). In a series of 69 CC, Xiaoying Liu et al. found a relation between down-regulation of miRNA-543 and LNM+ (12). Interestingly, we noted that two authors reported a douwnregulation of miRNA-143 correlated with LNM+ justifying further evaluation (20, 24).

### miRNA expression in CC biofluids samples

When considering CC onset and progression, the role of HPV has been highlighted demonstrating increased EGFR levels secondary to HPV16E6/E7 and the link with let-7i-5p, miRNA-181a-2-3p (61, 62).

In a series of 182 CC and 12 healthy controls, Wei Jiang et al. noted that low miRNA-101 serum level was associated with LNM+ (p=0.001) (63). Similarly, P. Liu et al. evaluated the serum miRNA-196a by qRT-PCR in 105 CC patients, 86 CIN patients, and 50 healthy controls (64). They demonstrated that serum miRNA-196a levels were higher in CC patients (p< 0.01) and CIN (p< 0.05) compared to healthy controls. Moreover, serum miRNA-196a was associated with LNM+ (p*=*0.018). Chen-Fei Zhou et al. also demonstrated that miR-221-3p overexpression was correlated with peritumoral lymphangiogenesis and LNM+ status (14). Moreover, they investigated whether miR-221-3p was detected in peripheral plasma of CC patients. In a subpopulation of 40 patients with stage I–II CC (20 with LNM+ and 20 with LNM-) an overexpression of exosomal miRNA-221-3p was observed in patients with LNM+ suggesting its relevance as a potential new biomarker of lymph node status. Comparing CC patients with and without LNM, Zhang Liang et al, observed a low miRNA-378a-3p serum expression in patients LNM+ (p=0.022) (65). Moreover, Qiu et al. have proposed a circulating miRNA signature for the diagnosis and prognosis of early-stage CC based on a series of 112 CC patients, 45 patients with CIN and 90 healthy subjects (66). They found a relation between LNM+ and high serum miRNA-21 associated with low serum miRNA-125b and miRNA-370. Among 17 patients with this signature the rate of LNM+ was 53% while for the 21 patients with simultaneously low serum miRNA-21 and high serum miRNA-125b and high serum miRNA- 370, none exhibited LNM. Although all aforementioned studies confirm the relation between some miRNAs and LNM status, all failed in determining a usable threshold in current practice.

More interesting, in a cohort of 80 patients with FIGO stages I-IIA CC, Zhao et al. (67) analyzed the expression of miRNA-20a and miRNA-203 in serum collected before surgery and treatment. Serum miRNA-20a was higher in patients with LNM+ with an area under the curve (AUC) of 0.734 ± 0.058, sensitivity of 75%, specificity of 72.5%, and a cut-off value of 3.0 as a marker for metastasis. Serum miRNA-203 was less relevant with an AUC of 0.658 ± 0.061, sensitivity of 65%, specificity of 62.5%, and a cut-off value of 0.13 (67). In a series of 100 patients (40 CC patients with LNM+, 40 CC patients with LNM-, and 20 healthy controls), among 89 miRNAs, J. Chen et al. (68) observed that 22 were upregulated and none downregulated. Using qRT-PCR, a panel of five miRNAs (miRNA-1246, miRNA-20a, miRNA-2392, miRNA-3147, miRNA-3162-5p, and miRNA-4484) was predictive of LNM with an AUC of 0.932, sensitivity of 85.6%, and specificity of 85.0%. For miRNA in tissue, the respective AUC, sensitivity and specificity were 0.992, 96.7%, and 95.0%. Moreover, on the same serum samples, the authors evaluated the relevance of SCC antigen as a predictor for lymph node metastasis showing a low relevance compared to the miRNA panel with an AUC of 0.713, sensitivity of 61.2%, and specificity of 70.0%. As previously reported by Zhao et al, these results confirm the relevance of serum miRNA-20a as a potential marker of lymph node status (67).

## CircRNA

CircRNA is a class of ncRNAs characterized by covalently closed loop without any 5’-3’ polarity or a polyadenylated tail (69). Thanks to New Generation Sequencing (NGS) and bioinformatic, more circRNAs have been identified (70) demonstrating their implication in both physiological and pathological processes such tumor cell proliferation, invasion and metastasis in various cancers (71). CircRNA regulate gene expression mainly as ceRNAs at various levels; epigenetic, transcriptional and post-transcriptional levels of protein-coding mRNAs (72–74).

A recent preliminary study in three HPV16 positive cervical cancer cases identified 99 deregulated circRNAs (58 over expressed and 41 under expressed circRNAs) (75). However, the data are too scant to evaluate the interaction between HPV and circ-RNAs in CC.

Using GSE30656 and TCGA, among 156 miRNAs, 5,321 mRNAs, and 75 circRNAs, Yuexiong Yi et al. identified regulatory circRNA-miRNA-mRNA networks with 7 hubgenes (RRM2, CEP55, CHEK1, KIF23, RACGAP1, ATAD2 and KIF11) implicated in CC (76). These hubgenes network including 5 circRNAs (circRNA-000596, circRNA-104315, circRNA-400068, circRNA-101958 and circRNA-103519) play a crucial role in chemosensitivity. In contrast to miRNAs expression in CC, little is known on the relation between circRNA expression and LNM (77). In a series of 68 CC, Huang Ma et al. noted an upregulation of circ-0005576 associated with LNM+ by sponging miR-153-3p (78). Recently, Tie-Fang Song et al. reported that upregulated circRNA-101996, binding to miR-1236-3p, was associated to LNM+ (p=0.038) (79). More interesting, in a series of 25 CC, Guo et al. have evaluated the interaction between circ-0023404 and miRNA-5047 showing an upregulation of circ-0023404 correlated to lymphatic angiogenesis associated with VEGFA upregulation (r=0.5448, p=0.0049). and downregulation of miRNA-5047 (r=0.7159, p<0.0001). The authors noted that circ-0023404 by regulating miRNA-5047 enhances metastasis and contributes to chemoresistance to cisplatin (80).

## LncRNA and cervical cancer

lncRNAs are now recognized as playing crucial roles in numerous cellular processes, including cell cycle (81), differentiation (82), metabolism (83), and in disease (84). lncRNAs can modulate transcription, epigenetic modifications, protein/RNA stability, translation, and posttranslational modifications by interacting with DNA (85), RNAs (86) and/or proteins (87). While lncRNAs do not encode protein, most of them lncRNAs are transcribed by RNA polymerase II (Pol II) and are capped and polyadenylated (88). Moreover, most lncRNAs are stabilized through polyadenylation (89). Although, lncRNA expression levels are typically lower than that of mRNAs (90), they display stronger tissue-specific expression patterns, suggesting integral roles in cell type–specific processes (91, 92). As proteins, lncRNAs act in different subcellular compartments by direct local molecular interactions maintaining cellular homeostasis.

Furthermore, recent studies have underline the potential distinction between HPV positive and negative CC in terms of prognostic. Indeed, Liu et al. in their review on lncRNAs stated HPV-negative cervical cancers are more likely diagnosed at non-squamous type, older ages, more advanced stage and metastases, and associated with poorer prognosis as compared to HPV-positive cervical cancer (93). All these data could justify combined evaluation of lncRNAs and HPV to evaluate their prognostic relevance.

### lncRNA expression in CC tissue samples

lncRNAs are involved in multiple physiological pathways and in cancer development including a role in CC progression, invasion and metastasis. In a review on lncRNA in CC, Tornesello et al. reported that HOTAIR, H19, MALAT1, CCAT2 SPRY4-IT1, CCHE1, PVT1, LINC00675, C5orf66-AS1, FAM83H-AS1, CCAT1, NOC2L-4.1, RSU1P2 have an oncogenic function while EBIC MEG3 GAS5, LET, PAX8 AS1 act as tumor suppressor (77).

In a meta-analysis on HOTAIR including six series with 535 patients, Shasha Liu et al. found that for the three series evaluating the relation between HOTAIR expression and LNM+, a high expression of HOTAIR was associated with positive lymph node status with an OR of 7.52 (95% CI: 3.72–15.20; *P* < 0.00001) suggesting its relevance as a new biomarker (94). Although analysis of I2 heterogeneity showed that p = 0.401, I2 = 0%, it is important to underlined that among the 365 patients with CC, lymph node status was available in only 180 cases (49.3%) and that HOTAIR expression analysis was performed using the median value compared to normal cervical tissue in two of the three series. Ping Chen et al. found that a high expression of lncRNA TTN-AS1 acting by sponging miRNA-573 was associated with LNM+ (95). In a series of 92 CC, Chun-Ling Yu et al. observed that high LINC00511 was associated with LNM+ (p<0.001) (96). In a series of 39 CC including 19 patients with pelvic LNM, Chunliang Shang et al. found 234 lncRNAs differentially expressed according to LNM status. LncRNA MIR100HG and AC024560.2 exhibited a respective AUC of 0.801 and 0.837 (97) (**Table 2**).

**Table 2:** Series evaluating the correlation between lncRNAs and lymph nodes invasion in patients with cervical cancer.

Author	Long non coding RNA	Down/Up regulated	Number of CC	LNM -	LNM +	p-value
Hee Jung Kim (98)	HOTAIR	–	111	19	32	0.73
Long Huang (99)	HOTAIRE	–	218	68	41	<0.001
Ping Chen (95)	TTN-AS1	–	45	28	17	0.008
Churliang Shang (100)	LNMICC	UP	211	20	47	0.0003
Churliang Shang (97)	MIR100HG AC024560.2	–	39	20	19	<0.01
Weichum Cao (101)	ATB	UP	187	127	60	< 0.01
Hui Liu (102)	LINC00861	DOWN	56	41	15	0.035
X-J Lv (103)	PCAT6	UP	114	83	31	0.028
Y-M Hou (104)	ZNF281	UP	58	36	22	0.018
YF Wang (105)	HULC	UP	244	133	111	< 0.001
Ben Di Mao (106)	LINC00511	UP	84	79	5	0.02
DW Wang (107)	LINC00518	UP	113	96	37	0.007
Chen Chen (108)	CCLM	DOWN	42	–	–	<0.05
Yue Zhong (109)	LINC00636	UP	59	44	15	<0.01
Yao Zhang (110)	ARAP1-AS1	UP	86	57	29	0.012

CC, Cervical cancer; LNM, Lymph node metastasis.

From the theragnostic point of view, Mao et al. reported an upregulated expression of LIN00511 in CC with LNM+ associated with paclitaxel (PTX) resistance. Moreover, they observed a relation between LIN00511 and multidrug resistance protein 1 (MRP1) and P-glycoprotein (P-GP) with potential therapeutic target by silencing LINC00511 (106).

### lncRNA expression in CC biofluids samples

In a series of 284 patients, Xian-zhen Ding et al. evaluated serum exosomal lncRNA DLX6-AS1 in 114 patients with CC, 60 patients with CIN and 110 healthy women (111). Serum exosomal lncRNA DLX6-AS1 level was significantly elevated in CC patients compared with CIN patients and healthy controls. Based on the median serum value, CC subjects were divided into high and low exosomal lncRNA DLX6-AS expression. High serum exosomal lncRNA DLX6-AS1 levels were correlated with LNM+ (p=0.0071). In addition to a high tissue expression of LNMICC with prometastatic effects suppressed by miR-190 in CC, Chunliang Shang et al. observed a relation between a high serum LNMICC expression and LNM+ (p=0.0003) (97). Similarly, Yue Zhong et al. observed both an overexpression of LINC00636 in tissue samples and in serum with a correlation with LNM+ (p<0.001) (109). In addition to tissue analysis of ARAP1-AS1 in 86 CC patients, Yao Zhang et al. compared serum ARAP1-AS1 expression in 37 CC patients and 20 healthy controls and observed an overexpression with an AUC value of 0.8953 (95% CI: 0.8147-0.9758, p< 0.001) (110). Moreover, serum ARAP1-AS1 expression was correlated with LNM+ (p=0.012) with HR of 2.161 (95% CI:1.354-3.148, p=0.036) (**Table 3**).

**Table 3:** 2018 FIGO classification of the uterine cervical cancer (112).

Stage	Description
I	The carcinoma is strictly confined to the cervix (extension to the uterine corpus should be disregarded)
IA	Invasive carcinoma that can be diagnosed only by microscopy, with maximum depth of invasion ≤5 mm^a^
IA1	Measured stromal invasion ≤3 mm in depth
IA2	Measured stromal invasion >3 and ≤5 mm in depth
IB	Invasive carcinoma with measured deepest invasion >5 mm (greater than Stage IA); lesion limited to the cervix uteri with size measured by maximum tumor diameter^b^
IB1	Invasive carcinoma >5 mm depth of stromal ivasion and ≤2 cm in greatest dimension
IB2	Invasive carcinoma >2 and ≤4 cm in greatest dimension
IB3	Invasive carcinoma >4 cm in greatest dimension
II	The carcinoma invades beyond the uterus, but has not extended onto the lower third of the vagina or to the pelvic wall
IIA	Involvement limited to the upper two-thirds of the vagina without parametrial involvement
IIA1	Invasive carcinoma ≤4 cm in greatest dimension
IIA2	Invasive carcinoma >4 cm in greatest dimension
IIB	With parametrial involvement but not up to the pelvic wall
III	The carcinoma involves the lower third of the vagina and/or extends to the pelvic wall and/or causes hydronephrosis or nonfunctioning kidney and/or involves pelvic and/or para-aortic lymph nodes
IIIA	The carcinoma involves the lower third of the vagina, with no extention to the pelvic wall
IIIB	Extension to the pelvic wall and/or hydronephrosis or nonfunctioning kidney (unless known to be due to another cause)
IIIC	Involvement of pelvic and/or para-aortic lymph nodes (including micrometastases)^c^, irrespective of tumor size and extent (with r and p notations)^d^
IIIC1	Pelvic lymph node metastasis only
IIIC2	Para-aortic lymph node metastasis
IV	The carcinoma has extended beyond the true pelvis or has involved (biopsy proven) the mucosa of the bladder or rectum. A bullous edema , as such, does not permit a case to be allotted to Stage IV
IVA	Spread of the growth adjacent pelvic organs
IVB	Spred to distant organs

## Perspectives

From the analysis of the literature on ncRNAs in tissue and biofluids in patients with CC, it appears that there are arguments to support their role in physiopathology, in the differential diagnosis from normal tissue, preinvasive and invasive tumors. However, these analyzes were mainly performed by microarray with validation by qRT-PCR with potential biases linked to the methodology such as the comparison between tumoral sample and adjacent normal appearance tissue and by the preselection of some miRNAs as proven in other context (113). In addition, most studies are from China raising the issue on variations of HPV genotypes according to ethnicity thus on ncRNA expression (114). Moreover, the relatively small number of studies with limited sample size focusing on early CC stages especially concerning miRNAs expression in biofluids of patients with CC limit their potential clinical utility. Indeed, as previously underlined (115) miRNAs are abundant in tissues but often rare in plasma and serum. Moreover, it has been also pointed out that for the quantification of miRNAs in plasma, as well as in other biofluids such as urine and saliva, there is a need to use high-sensitivity platforms such as Next Generation Sequencing (NGS) and Bioinformatic. So far, whatever tissue or biofluids samples, no study using NGS and bioinformatic has been published to support their role in the management of CC. However, all these studies underlined a true relation between miRNA, circRNA and lncRNA with LNM status with a potential non-invasive signature and a relation with response to neo- and adjuvant therapies with a potential contribution in a new algorithm to manage cervical cancer.

## Author contributions

Methodology and Design: YD, CT, ED, SB. Data collection: YD, AF, LR, YM. Analysis: YD, CF, SS, AF. Data Interpretation: YD, LR, CF, SB, CT. Manuscript writing: AF, SS, CT, YM. All authors reviewed the manuscript for critical intellectual content. All authors contributed to the article and approved the submitted version.
